# Genome-wide association and prediction of direct genomic breeding values for composition of fatty acids in Angus beef cattle^a^

**DOI:** 10.1186/1471-2164-14-730

**Published:** 2013-10-25

**Authors:** Mahdi Saatchi, Dorian J Garrick, Richard G Tait, Mary S Mayes, Mary Drewnoski, Jon Schoonmaker, Clara Diaz, Don C Beitz, James M Reecy

**Affiliations:** 1 Department of Biochemistry, Biophysics and Molecular Biology, Iowa State University, Ames, IA 50011, USA; 2 Department of Animal Science, Iowa State University, 2255 Kildee Hall, Ames, IA 50011, USA; 3 Department of Animal Science, Purdue University, West Lafayette, IN 47907, USA; 4 INIA, Depto. de Mejora Genética Animal, Ctra. de La Coruña Km 7.5, Madrid 28040, Spain; 5 Department of Animal and Veterinary Science, University of Idaho, Moscow, ID 83844, USA; 6 Institute of Veterinary, Animal and Biomedical Sciences, Massey University, Palmerston North 4442, New Zealand; 7 Present address: USDA, Agricultural Research Service, U.S. Meat Animal Research Center, Clay Center, NE 68933, USA

**Keywords:** Intramuscular fat, Genome architecture, Angus

## Abstract

**Background:**

As consumers continue to request food products that have health advantages, it will be important for the livestock industry to supply a product that meet these demands. One such nutrient is fatty acids, which have been implicated as playing a role in cardiovascular disease. Therefore, the objective of this study was to determine the extent to which molecular markers could account for variation in fatty acid composition of skeletal muscle and identify genomic regions that harbor genetic variation.

**Results:**

Subsets of markers on the Illumina 54K bovine SNPchip were able to account for up to 57% of the variance observed in fatty acid composition. In addition, these markers could be used to calculate a direct genomic breeding values (DGV) for a given fatty acids with an accuracy (measured as simple correlations between DGV and phenotype) ranging from -0.06 to 0.57. Furthermore, 57 1-Mb regions were identified that were associated with at least one fatty acid with a posterior probability of inclusion greater than 0.90. 1-Mb regions on BTA19, BTA26 and BTA29, which harbored fatty acid synthase, Sterol-CoA desaturase and thyroid hormone responsive candidate genes, respectively, explained a high percentage of genetic variance in more than one fatty acid. It was also observed that the correlation between DGV for different fatty acids at a given 1-Mb window ranged from almost 1 to -1.

**Conclusions:**

Further investigations are needed to identify the causal variants harbored within the identified 1-Mb windows. For the first time, Angus breeders have a tool whereby they could select for altered fatty acid composition. Furthermore, these reported results could improve our understanding of the biology of fatty acid metabolism and deposition.

## Background

In response to the constant bombardment of health-related stories, consumers are becoming more health conscious and are becoming increasingly aware of the amount and type of fats and fatty acids they consume. Red meat is often perceived as a fatty protein source with certain health risks associated with its consumption. Beef could be viewed more favorably from a human health standpoint if strategies could be applied to decrease saturated fatty acid (SFA) content while increasing the concentration of beneficial polyunsaturated fatty acids (PUFA), especially omega-3 PUFA, and conjugated linoleic acid.

Beef producers continue to strive to produce a high quality product that meets consumer demands in a cost-effective manner. While fatty acid profiles can be altered through the diet [1,2], identification of genetic markers that would allow producers to select beef for altered fatty acid composition could ultimately increase value and consumer satisfaction with beef. While producers have recently selected cattle with a higher propensity to marble, because of the premiums that they are paid, some consumers favor lower concentrations of SFA because of their perceived negative effect on human health. Therefore, the goal of the present study was to assess the utility of genetic markers to select for fatty acids composition, identify regions of the genome that account for genetic variation, and evaluate genome architecture of fatty acid regulation.

## Results and discussion

Summary statistics for the fatty acid phenotypes analyzed in this study are reported in Table 1.

**Table 1:** **The summary statistics of mean (***μ***), standard deviation (SD) and coefficient of variation (CV) for all studied fatty acids traits in both meat and fat percent bases**

	**Beef meat basis**^**1**^			**Fat percent basis**^**2**^		
**Trait**	*μ*, g × 10^- 5^	*SD*, g × 10^- 5^	*CV* × 100	*μ*, %	*SD*, %	*CV* × 100
10:0	1.96	2.72	138.3	0.035	0.049	138.3
12:0	3.59	3.38	94.2	0.062	0.055	88.6
13:0	0.27	0.57	215.7	0.005	0.010	213.7
14:0	160.34	73.46	45.8	2.707	0.574	21.2
14:1	33.32	17.45	52.4	0.565	0.196	34.6
15:0	33.84	18.79	55.5	0.593	0.330	55.7
16:0	1,558.61	596.70	38.3	26.549	1.792	6.7
16:1	206.06	92.83	45.0	3.478	0.710	20.4
17:0	81.07	42.57	52.5	1.347	0.392	29.1
17:1	64.24	34.59	53.8	1.071	0.369	34.4
18:0	790.43	292.08	37.0	13.637	1.887	13.8
*cis-*9 18:1	2,281.82	923.99	40.5	38.555	2.787	7.2
*cis-*11 18:1	5.89	6.93	117.7	0.099	0.105	106.0
*cis-*12 18:1	15.59	13.05	83.7	0.255	0.162	63.8
*cis-*13 18:1	5.87	7.47	127.2	0.097	0.103	106.4
*trans-*6/9 18:1	8.09	12.48	154.1	0.128	0.19	148.5
*trans*-10/11 18:1	212.59	119.07	56.0	3.599	1.38	38.3
*trans*-12 18:1	3.98	10.09	253.4	0.063	0.128	202.4
*trans*-15 18:1	61.99	39.95	64.4	1.037	0.506	48.8
18:2	217.59	70.66	32.5	3.948	1.313	33.3
18:3n3^3^	10.52	11.19	106.3	0.171	0.158	92.3
18:3n6^4^	0.88	2.32	263.8	0.014	0.033	227.7
20:0	1.10	1.95	177.0	0.020	0.034	170.2
20:1	4.88	5.99	122.8	0.094	0.110	117.1
20:2	2.07	2.87	138.6	0.037	0.048	132.3
20:3n3^3^	1.49	5.48	368.9	0.024	0.093	380.6
20:3n6^4^	7.25	8.88	122.4	0.122	0.154	126.3
20:4	41.38	16.37	39.5	0.773	0.378	48.9
20:5	6.80	12.89	189.5	0.133	0.282	212.2
22:0	5.45	7.03	129.1	0.110	0.152	137.7
22:1	0.30	3.20	1,079.0	0.005	0.056	1,107.1
22:4	3.18	6.71	211.3	0.062	0.135	216.6
22:5	7.5	8.81	117.6	0.130	0.162	124.6
22:6	4.02	7.43	185.0	0.083	0.161	193.9
23:0	3.54	8.11	229.0	0.069	0.170	244.9
24:0	7.27	17.32	238.1	0.143	0.367	257.2
CLAc9t11	7.32	8.29	113.3	0.125	0.127	101.2
CLAt10c12	3.32	5.06	152.6	0.051	0.071	138.3
MCFA	233.32	100.00	42.9	3.967	0.785	19.8
LCFA	5,632.1	2,098.75	37.3	96.033	0.785	0.8
MUFA	2,940.62	1,168.08	40.2	49.047	2.795	5.7
PUFA	313.31	101.58	32.4	5.674	1.849	32.6
SFA	2,647.48	976.97	36.9	45.279	2.384	5.3
PUFA/SFA	NA^6^	NA	NA	12.6	4.285	34.0
(14:0+16:0)/All	NA	NA	NA	29.257	2.197	7.5
Al^5^	NA	NA	NA	68.728	8.856	12.9
Σ n-3 fatty acids	30.33	28.3	93.3	0.541	0.536	99.0
Σ n-6 fatty acids	282.99	88.41	31.2	5.134	1.648	32.1
n3/n6	NA	NA	NA	10.933	12.836	117.4

^1^The amount of fatty acid in 1 gram beef meat.

^2^The percent of fatty acid in total fatty acid.

^3^n-3 fatty acids.

^4^n-6 fatty acids.

^5^Atherogenic Index.

^6^NA = Not Analyzed.

### Posterior genetic and residual variances and heritability

The discovery process generates an estimate, similar to pedigree-based heritability, of the proportion of phenotypic variation that can be accounted for using SNP markers for each of the fatty acids studied on a beef meat or fat percent basis (Table 2). The proportion of phenotypic variance explained (h^2^) by SNP genotypes varied from a very low amount (0.06) for 18:1c13, 18:1t6pt9, 18:3n6, and 20:3n3, which indicates that the marker predictions will be poor, to relatively high (> 0.49) for 14:0, 14:1,16:0, 16:1, 18:0, 18:1c9 and 24:0, which indicates potential for relatively good marker predictions. In general, the percentage of phenotypic variance explained by markers was higher when fatty acids were analyzed on a fat percent compared with beef basis. This result is not unexpected given that, on a beef basis, the level of any given fatty acid is influenced by both its relative amount in comparison to other fatty acids as well as the amount of lipid present in the given sample. In contrast, on a fat percent basis, only variation relative to other fatty acids is taken into account. If total fatty acid content is included as a covariate when analyzing fatty acids on a beef basis, heritabilities similar to a weight percent basis are obtained (data not shown). This comparison would indicate that much of the variation in heritability estimates between methods result from variation in total fatty acid content. On a fat percent basis, fatty acids with chain length >18 carbons (with the exception of 24:0), had lower heritability (0.06 to 0.24) than shorter chain fatty acids (0.08 to 0.57). This heritability difference might indicate that genes involved in the production and/or metabolism of these longer-chain fatty acids are under selective pressure to minimize variation. Alternatively, given the fact that *de novo* fatty acid synthesis in cattle is limited to primarily 14, 16 and 18 carbon fatty acids [3], it is possible that the observed variation in longer chain fatty acids result from host genetic variation influencing the population of rumen microbiota, which modify ingested fatty acids [4,5].

**Table 2:** **The posterior estimates of genetic**$\left(\sigma_{g}^{2}\right)\;$**and residual**$\left(\sigma_{e}^{2}\right)$**variances, and the estimated heritability** (*h*^2^) **for all studied fatty acids traits in both meat and fat percent bases**

	**Beef meat basis**^**1**^			**Fat percent basis**^**2**^		
**Trait**	$\sigma_{g}^{2},g\times 10^{-10}$	$\sigma_{e}^{2},g\times 10^{-10}$	*h*^2^	$\sigma_{g}^{2},{\%}^{2}$	$\sigma_{e}^{2},{\%}^{2}$	*h*^2^
10:0	0.46	5.06	0.08	0.020	0.161	0.11
12:0	0.69	8.34	0.07	0.021	0.230	0.08
13:0	0.02	0.21	0.08	0.002	0.006	0.23
14:0	545.12	965.70	0.36	15.039	10.973	0.57
14:1	39.33	75.18	0.34	1.286	1.239	0.50
15:0	17.59	129.14	0.11	0.640	4.757	0.11
16:0	9,413.38	36,901.10	0.20	123.728	114.915	0.51
16:1	696.82	1,523.51	0.31	20.838	21.594	0.49
17:0	105.67	293.53	0.26	2.038	3.745	0.35
17:1	55.36	210.98	0.20	1.177	3.459	0.25
18:0	4,489.89	12,004.10	0.27	109.657	100.044	0.52
*cis-*9 18:1	25,140.30	87,427.20	0.22	309.422	246.831	0.55
*cis-*11 18:1	4.16	32.42	0.11	0.096	0.757	0.11
*cis-*12 18:1	17.85	58.20	0.23	0.409	1.159	0.26
*cis-*13 18:1	3.52	40.93	0.07	0.061	0.846	0.06
*trans-*6/9 18:1	7.07	91.38	0.07	0.229	2.190	0.09
*trans*-10/11 18:1	1,565.02	4,165.66	0.27	49.360	73.082	0.40
*trans*-12 18:1	7.44	81.36	0.08	0.198	1.195	0.14
*trans*-15 18:1	115.44	666.15	0.14	2.484	14.401	0.14
18:2	461.72	2,066.77	0.18	21.342	72.984	0.22
18:3n3^3^	9.45	47.31	0.16	0.161	0.945	0.14
18:3n6^4^	0.31	3.60	0.07	0.007	0.070	0.08
20:0	0.27	2.14	0.11	0.007	0.058	0.11
20:1	1.37	10.31	0.11	0.038	0.270	0.12
20:2	0.52	5.99	0.08	0.015	0.171	0.07
20:3n3^3^	1.48	21.58	0.06	0.043	0.644	0.06
20:3n6^4^	4.58	39.74	0.10	0.162	1.277	0.11
20:4	23.44	140.67	0.14	1.159	6.833	0.14
20:5	27.36	83.19	0.24	1.136	4.405	0.20
22:0	1.69	12.64	0.11	0.087	0.823	0.09
22:1	0.88	9.37	0.08	0.030	0.284	0.09
22:4	3.79	22.87	0.14	0.194	0.965	0.16
22:5	4.80	28.16	0.14	0.177	1.036	0.14
22:6	8.23	25.38	0.24	0.367	1.162	0.24
23:0	4.54	42.02	0.09	0.239	1.869	0.11
24:0	119.67	87.27	0.57	4.563	4.366	0.51
CLAc9t11	4.60	36.89	0.11	0.116	0.896	0.11
CLAt10c12	2.12	13.47	0.13	0.041	0.279	0.12
MCFA	933.11	1,799.59	0.34	26.700	21.357	0.55
LCFA	89,165.30	40,2578.00	0.18	26.661	21.375	0.55
MUFA	33,849.00	130,548.00	0.20	239.263	246.074	0.49
PUFA	789.96	3,981.50	0.16	35.037	144.961	0.19
SFA	23,398.20	94,644.80	0.19	243.243	183.208	0.57
PUFA/SFA	NA^6^	NA	NA	222.28	805.27	0.21
(14:0+16:0)/All	NA	NA	NA	206.16	172.13	0.54
Al^5^	NA	NA	NA	3,728.57	2,699.21	0.58
Σ n-3 fatty acids	102.78	266.89	0.27	4.270	10.717	0.28
Σ n-6 fatty acids	624.36	3,230.87	0.16	28.776	117.177	0.19
n3/n6	NA	NA	NA	1,397.28	9,703.89	0.12

^1^The amount of fatty acid in 1 gram beef meat.

^2^The percent of fatty acid in total fatty acid.

^3^n-3 fatty acids.

^4^n-6 fatty acids.

^5^Atherogenic Index.

^6^NA = Not Analyzed.

Medium-chain saturated fatty acids like 12:0, 14:0 and 16:0 have been associated with increased incidence of cardiovascular disease [6,7]). In contrast, longer-chained and unsaturated fatty acids are considered to be either neutral or even possibly protective [8-10].

Given the relatively high amount of phenotypic variance in 14:0, 16:0, 18:0, 18:1c9, (14:0 + 16:0)/All and AI that variation that can be accounted for by molecular markers, it should be possible to select for a more heart healthy fatty acid composition.

### Direct genomic breeding values (DGV) coefficients, correlations and accuracy

The numbers of individuals in each K-means clustered group are shown in Table 3. The pooled regression coefficients and the simple correlations between DGV and phenotypes over 5 K-means clustered groups, and the realized accuracies of DGV for some fatty acid traits are in Table 4. The pooled regression coefficient ranged from 1.53 for CLAc9t11 to -0.47 for 20:3n3, the pooled simple correlation ranged from 0.43 for 14:0, MCFA, and AI to -0.02 for 20:3n3, while the accuracies of genomic prediction varied from 0.57 for 14:0, LCFA, and MCFA to -0.06 for 20:3n3 (Table 4). Given the higher accuracies associated with 14:0 and 16:0, it should be possible to develop a selection index to minimize these two fatty acids. Alternatively, producers could use ratios like (14:0 + 16:0)/All or AI to select for animals that have decreased levels of shorter chain saturated fatty acids.

**Table 3:** The number of individuals in each K-means clustered groups

Groups	1	2	3	4	5
Number	628	486	407	393	196^1^

^1^Combined from two primarily K-means clustered groups with sizes of 158 and 38.

**Table 4:** **The pooled regression coefficient of phenotype on DGV (***b*_(*P*,*DGV*)_)**, the pooled simple correlation between DGV and phenotype (***r*_(*DGV*,*P*)_**), and the realized accuracy**^**1 **^**of DGV for some fatty acid traits as percent in total fatty acid**

**Trait**	***b***_**( *P *, *DGV *)**_	***r***_**( *DGV *, *P *)**_	**Accuracy**
14:0	0.93	0.43	0.57
16:0	0.95	0.38	0.53
18:0	0.66	0.20	0.27
*cis-*9 18:1	0.77	0.26	0.35
*cis-*12 18:1	0.89	0.18	0.35
*trans*-12 18:1	0.03	0.01	0.03
18:3n3	0.13	0.01	0.03
18:3n6	0.22	0.06	0.21
20:3n3	-0.47	-0.02	-0.06
20:3n6	-0.04	-0.01	-0.02
20:4	0.03	0.00	0.01
CLAc9t11	1.53	0.10	0.29
CLAt10c12	0.04	0.00	0.01
LCFA	0.95	0.42	0.57
MCFA	0.95	0.43	0.57
MUFA	0.87	0.26	0.38
PUFA	0.37	0.04	0.08
SFA	0.84	0.34	0.45
PUFA/SFA	0.45	0.06	0.12
(14:0+16:0)/All	0.94	0.40	0.55
Al	0.92	0.43	0.56
n3	-0.01	-0.01	-0.01
n6	0.18	0.02	0.07
n3/n6	0.00	0.00	0.00

^1^As the pooled simple correlations between DGV and phenotypes in validation groups divided by the square root of trait heritability.

### Whole genome association

The 1-Mb SNP windows with the highest genetic variances and a posterior probability of having non-zero genetic variance greater than 90% (PPI) for fatty acids on a fat percent (Table 5) and beef basis (Table 6), respectively. The proportion of genetic variance explained by 1-Mb SNP windows ranged from 78.6% for 18:3n6 to 1.6% for 24:5 (Table 5) on a fat percent basis, and 60.5% for 10:0 and 1.5% for 24:0 on a beef basis (Table 6). Many of the 1-Mb windows were associated with more than one fatty acid. For example, the 51^st^ Mb window on chromosome 19 was associated with 14:0, 14:1, 16:0, 16:1, 18:1c9, LCFA, MCFA, MUFA, SFA, (14:0+16:0)/All, and AI on a fat percent basis. Whereas, only the 49^th^ Mb on chromosome 24 was associated with 17:1 (Table 5). No other region on chromosome 24 was associated with any other fatty acid.

**Table 5:** The 1-Mb SNP windows with the highest genetic variances and the posterior probability of having non-zero genetic variance greater than 90% for fatty acid traits on a fat percent basis

**Trait**	**BTA_Mb**^**1**^	**Start SNP**	**End SNP**	**Number of SNP**	**Genetic variance (%)**	**PPI**^**2**^
13:0	15_60	rs41662110	rs81159430	23	33.8	1
	19_20	rs110752559	rs109057891	20	15.0	1
14:0	19_51	rs41923412	rs109147235	25	37.8	1
	29_18	rs42375315	rs43770775	14	17.1	1
	10_19	rs41647457	rs110785951	24	6.2	1
	26_21	rs109309604	rs42086690	20	4.6	0.998
	19_53	rs110146710	rs41577620	25	3.4	0.992
	6_109	rs43486482	rs43483949	24	2.3	0.950
14:1	19_51	rs41923412	rs109147235	25	22.1	1
	29_18	rs42375315	rs43770775	14	14.0	1
	10_19	rs41647457	rs110785951	24	12.0	1
	26_21	rs109309604	rs42086690	20	11.0	0.998
15:0	2_18	rs29009916	rs43293795	29	42.1	0.985
	1_134	rs109189105	rs110223085	22	14.4	1
16:0	19_51	rs41923412	rs109147235	25	28.8	1
	29_18	rs42375315	rs43770775	14	14.0	1
16:1	19_51	rs41923412	rs109147235	25	15.6	1
	29_18	rs42375315	rs43770775	14	8.0	1
	26_21	rs109309604	rs42086690	20	7.6	1
	10_19	rs41647457	rs110785951	24	5.3	0.999
17:0	26_33	rs41606739	rs110568468	27	5.8	0.904
	19_43	rs41915671	rs109729658	19	5.5	0.928
17:1	24_49	rs110838391	rs41585203	14	10.6	0.939
18:0	29_18	rs42375315	rs43770775	14	11.2	1
*cis-*9 18:1	19_51	rs41923412	rs109147235	25	29.9	1
	29_18	rs42375315	rs43770775	14	6.7	1
	16_4	rs110257825	rs109105804	26	3.7	0.923
*cis-*11 18:1	28_20	rs42137452	rs43702480	21	29.1	0.985
*cis-*12 18:1	26_21	rs109309604	rs42086690	20	27.1	1
*trans-*6/9 18:1	1_64	rs110449758	rs43233287	26	53.9	1
	2_90	rs43703384	rs108939546	16	47.3	0.981
	1_84	rs41635181	rs43246311	23	26.6	1
	19_20	rs110752559	rs109057891	20	16.4	1
	2_66	rs109157575	rs41604324	15	12.4	1
*trans*-12 18:1	28_45	rs110589396	rs42157158	25	25.6	1
	20_39	rs110243640	rs110201922	28	13.4	0.947
*trans*-15 18:1	13_39	rs110560225	rs41692994	26	13.1	0.962
18:3n6	2_9	rs43289248	rs41564963	20	78.6	1
20:3n6	15_11	rs42812364	rs41661666	14	48.4	1
20:5	2_91	rs110681542	rs41598586	10	7.7	0.98
	9_59	rs110542333	rs41659809	29	2.7	0.978
	8_39	rs29011524	rs109724258	19	1.6	0.943
22:1	18_4	rs81168102	rs109801196	20	27.2	1
	10_56	rs43633230	rs42997789	22	18.6	1
	13_36	rs41583782	rs110257518	22	15.1	1
	17_36	rs41637570	rs110869626	16	11.3	1
	7_11	rs42975215	rs41630355	3	8.5	1
	21_52	rs43705682	rs43110731	24	7.0	0.987
	8_29	rs43547661	rs109569294	22	6.2	0.965
	29_24	rs43178042	rs29027373	23	4.9	0.968
	21_10	rs42827268	rs109582710	24	4.4	0.986
22:4	23_7	rs29013434	rs41642917	23	15	1
	9_59	rs110542333	rs41659809	29	13.0	1
	28_14	rs41648888	rs42135312	18	5.0	0.905
22:5	2_91	rs110681542	rs41598586	10	36.8	0.999
	27_35	rs41572913	rs109612018	23	8.2	0.960
22:6	15_56	rs42996690	rs109535431	22	27.0	0.999
	15_11	rs42812364	rs41661666	14	9.0	1
24:0	7_15	rs109570025	rs110440896	15	35.6	1
	7_45	rs110404881	rs41606984	18	24.3	1
	2_49	rs109941542	rs110991778	8	15.7	1
	2_132	rs109889085	rs110709504	17	7.1	0.998
	3_81	rs110827478	rs43351357	31	4.7	1
	19_37	rs109433582	rs110497942	22	3.8	0.935
	19_20	rs110752559	rs109057891	20	2.9	0.991
	9_3	rs43582937	rs41610313	15	1.8	0.975
	8_39	rs29011524	rs109724258	19	1.8	0.974
	17_46	rs41842253	rs109295315	26	1.7	0.917
LCFA	19_51	rs41923412	rs109147235	25	40.5	1
	29_18	rs42375315	rs43770775	14	15.7	1
	10_19	rs41647457	rs110785951	24	8.9	1
	10_18	rs110963111	rs109738686	25	3.8	0.979
	18_18	rs110528295	rs110871891	25	2.5	0.906
MCFA	19_51	rs41923412	rs109147235	25	39.7	1
	29_18	rs42375315	rs43770775	14	15.8	1
	10_19	rs41647457	rs110785951	24	9.1	1
	10_18	rs110963111	rs109738686	25	3.8	0.981
MUFA	19_51	rs41923412	rs109147235	25	21.9	1
	16_4	rs110257825	rs109105804	26	4.6	0.994
SFA	19_51	rs41923412	rs109147235	25	18.4	1
	26_21	rs109309604	rs42086690	20	7.0	0.998
	7_93	rs109819349	rs29009626	11	5.0	0.989
	1_115	rs41596623	rs43712701	20	4.6	0.998
	16_4	rs110257825	rs109105804	26	3.9	0.995
PUFA/SFA	7_93	rs109819349	rs29009626	11	9.4	0.920
(14:0+16:0)/All	19_51	rs41923412	rs109147235	25	29.7	1
	29_18	rs42375315	rs43770775	14	16.8	1
AI	19_51	rs41923412	rs109147235	25	29.6	1
	29_18	rs42375315	rs43770775	14	13.8	1
	26_21	rs109309604	rs42086690	20	4.7	1
	19_48	rs41918815	rs29025977	21	3.3	0.990
n3	16_63	rs41638728	rs42252603	20	68.0	1
	18_19	rs29009603	rs41660721	20	40.3	1
	7_45	rs110404881	rs41606984	18	39.4	0.991
	2_91	rs110681542	rs41598586	10	7.3	0.992
	15_11	rs42812364	rs41661666	14	7.2	0.995
n6	2_18	rs29009916	rs43293795	29	15.3	0.985
n3/n6	1_112	rs110853931	rs41573010	26	51.5	0.950
	15_56	rs42996690	rs109535431	22	31.1	1
	17_36	rs41637570	rs110869626	16	9.5	0.998
	1_21	rs41625140	rs109126050	23	5.9	0.999

^1^Bovine chromosome and n^th^ 1-Mb window of the same chromosome started from zero, based on UMD 3.1.

^2^Posterior probability of inclusion (non-zero genetic variance).

**Table 6:** The 1-Mb SNP windows with the highest genetic variances and the posterior probability of having non-zero genetic variance greater than 90% for fatty acid traits on a beef basis

**Trait**	**BTA_Mb**^**1**^	**Start SNP**	**End SNP**	**Number of SNP**	**Genetic variance (%)**	**PPI**^**2**^
10:0	15_65	rs111001091	rs110703505	28	60.5	1
	9_79	rs41568875	rs41594191	11	9.5	0.996
	4_56	rs43394097	rs41588642	21	5.8	0.900
13:0	19_20	rs110752559	rs109057891	20	22.5	0.977
14:0	19_51	rs41923412	rs109147235	25	23.2	1
	29_18	rs42375315	rs43770775	14	19.0	1
	26_21	rs109309604	rs42086690	20	5.0	0.967
14:1	19_51	rs41923412	rs109147235	25	15.6	1
	29_18	rs42375315	rs43770775	14	15.0	1
	10_19	rs41647457	rs110785951	24	11.1	1
15:0	22_41	rs42010046	rs41613651	23	15.6	0.937
16:1	29_18	rs42375315	rs43770775	14	8.5	1
	19_51	rs41923412	rs109147235	25	7.8	0.998
	26_21	rs109309604	rs42086690	20	7.1	1
	1_124	rs42904587	rs41610871	16	5.5	0.980
17:0	19_43	rs41915671	rs109729658	19	9.6	0.965
	29_19	rs42375315	rs43770775	14	7.3	0.903
18:0	29_18	rs42375315	rs43770775	14	11.0	1
*cis-*9 18:1	19_51	rs41923412	rs109147235	25	25.1	1
*cis-*12 18:1	26_21	rs109309604	rs42086690	20	24.1	1
*trans-*6/9 18:1	2_66	rs109157575	rs41604324	15	32.6	0.998
*trans*-12 18:1	28_45	rs110589396	rs42157158	25	25.2	1
	20_39	rs110243640	rs110201922	28	14.5	1
	5_26	rs109601171	rs110457668	15	13.3	0.977
*trans*-15 18:1	22_32	rs29019970	rs110288437	21	32.7	1
						
18:3n6	7_11	rs42975215	rs41630355	3	49.5	1
20:3n6	15_11	rs42812364	rs41661666	14	48.3	1
20:5	9_39	rs110362207	rs41657531	14	27.6	1
	15_11	rs42812364	rs41661666	14	7.1	0.988
22:1	10_88	rs42249704	rs42342704	27	36.1	1
	7_11	rs42975215	rs41630355	3	24.0	1
	17_9	rs41570593	rs41634896	24	13.9	1
	17_36	rs41637570	rs110869626	16	11.7	1
	13_36	rs41583782	rs110257518	22	10.9	1
	5_84	rs110074949	rs41616137	17	6.3	0.999
	8_29	rs43547661	rs109569294	22	4.7	0.914
	X_72	rs42201987	rs109917570	5	3.7	0.955
22:5	18_2	rs41858629	rs41854877	22	30.9	0.977
22:6	15_56	rs42996690	rs109535431	22	36.3	0.997
	15_11	rs42812364	rs41661666	14	13.7	1
	7_43	rs41614886	rs43512367	26	4.1	0.982
24:0	7_15	rs109570025	rs110440896	15	46.2	1
	29_49	rs109580937	rs110325032	23	30.8	1
	2_132	rs109889085	rs110709504	17	20.4	1
	21_14	rs110534906	rs109331211	20	11.3	0.97
	4_24	rs42604408	rs43379277	20	6.9	0.998
	2_49	rs109941542	rs110991778	8	6.5	1
	11_75	rs109520936	rs109636296	23	4.2	0.968
	3_81	rs110827478	rs43351357	31	3.6	1
	27_33	rs43733230	rs41590295	21	2.8	0.988
	17_46	rs41842253	rs109295315	26	2.3	0.997
	19_20	rs110752559	rs109057891	20	1.7	0.981
	4_66	rs109343093	rs109916601	25	1.5	0.987
MCFA	19_51	rs41923412	rs109147235	25	25.0	1
	29_18	rs42375315	rs43770775	14	17.8	1
MUFA	19_51	rs41923412	rs109147235	25	16.3	0.998
n3	8_70	rs110396523	rs42592620	23	45.0	1
	15_11	rs42812364	rs41661666	14	12.8	1
	13_2	rs109417988	rs41610896	26	2.8	0.909
n6	7_93	rs109819349	rs29009626	11	11.4	0.944

^1^Bovine chromosome and n^th^ 1-Mb window of the same chromosome started from zero, based on UMD 3.1.

^2^Posterior probability of inclusion (non-zero genetic variance).

Many of the 1-Mb windows that were identified harbored good candidate genes. For example, fatty acid synthase (FASN) is located on chromosome 19 between 51,384,922 and 51,403,614 bp, which is almost exactly in the middle of this 1-Mb window. Previously, our group reported that variants in FASN were associated with fatty acid composition in Angus [11]. In addition, FASN has been reported to be associated with bovine adipose composition, milk fat content, and fatty acid composition of beef in several different breeds of cattle, which indicates that it has a conserved role across genetic backgrounds [12-22]. Interestingly, there are several different variants that are responsible for FASN effects in the different breeds [11,12]. Furthermore, Sterol-CoA desaturase (SCD) is located on chromosome 26 between 21,132,751 and 21,133,969 bp, which is at the edge of a 1-Mb window associated with 14:0, 14:1, 16:1, *cis-*12 18:1, SFA, and AI (Tables 5 and 6). Previously, SCD variants have been reported to be associated with fatty acid composition of meat and milk fat [17,18,20-29]. In contrast, other 1-Mb regions contain no obvious candidate genes, for example the 20^th^ Mb window on chromosome 28 that is associated with *cis-*11 18:1. After the 1-Mb window that harbors FASN, a region on chromosome 29 (18^th^ Mb window) could account for the second greatest amount of genetic variance in 14:0, 14:1, 16:0, 16:1, *cis-*9 C18:1, LCFA, and MCFA. This region has not previously been reported to be associated with any adipose trait other than subcutaneous fat thickness (http://animalgenome.org/cgi-bin/QTLdb/BT/index) [30]. Interestingly, thyroid hormone responsive (THRSP) has been reported to act at the level of transcription to regulate genes that encode enzymes required for long-chain fatty acid synthesis [31]. In addition in knockout studies, it has been reported that THRSP null mice showed a marked deficiency in *de novo* lipogenesis. Moreover, knockout studies have also revealed that THRSP may work in the cytoplasm by tethering FASN to the microtubule [32]. Thus, it would appear that THRSP is a good candidate gene, which was recently reported to be associated with fatty acid composition in Korean cattle [33].

It should be noted that none of the 1-Mb windows that harbor SREBP1, ACACA, PPARG, FABP4, ACSL1, LEP, or LXRA, which are all genes that have been previously associated with fatty acid composition in beef [17,34-38], were associated with variation in any fatty acid in this study. When taken in concert with the fact that different FASN alleles appear to be segregating in different breeds [11,16], this may indicate that the genetic mechanisms controlling fatty acid composition may vary greatly from breed to breed. This is further supported by the fact that the FASN region in Japanese Black cattle appears to account for the vast majority of the genetic variance, while in contrast several regions are reported here for American Angus.

### Within regions correlation

**Figure 1:**
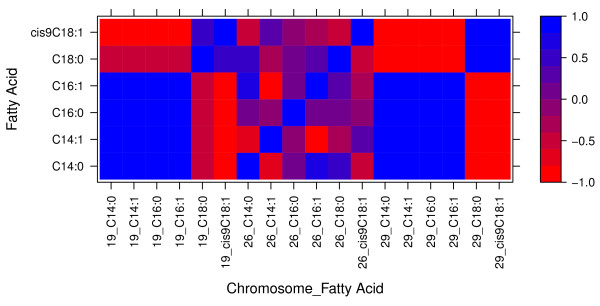
**Within 1-Mb region correlations of direct genomic breeding values for C14:0, C14:1, C16:0, C16:1, C18:0, and cisC918:1 fatty acids on a fat percent basis.** Regions are identified by chromosome number on the X-axis. The Y-axis represents the fatty acids for the same 1-Mb region on the X-axis.

The correlations between DGV within 19_51, 26_21 and 29_18 windows (windows harboring the candidate genes FASN, SCD and THRSP, respectively) for each pair of C14:0, C14:1, C16:0, C16:1, C18:0 and *cis-*9 C18:1 fatty acids on the fat percent basis are summarized in Figure 1. There are two clear patterns in the within windows estimated correlations between fatty acid. The first pattern involves the regions located on chromosome 19 (19_51) and 29 (29_18), which harbor FASN and THRSP as candidate genes, respectively. Estimates of the DGV correlations were very high and positive among 14:0, 14:1, 16:0 and 16:1, however regional DGV correlation between this group of fatty acids and 18:0 and *cis-*9 18:1 were large and negative. While the DGV correlation between 18:0 and *cis-*9 18:1 were very high and positive. Regions 19_51 and 29_18 were found to be associated to all fatty acids except for 18:0, where only the region on chromosome 29 was identified (Table 5). These results indicate that both, FASN and THRSP, exhibit pleiotropic effects for most fatty acids and act in a coordinate manner to contribute to the formation of fatty acid involved in *de novo* synthesis. However, for the formation of 18:0 and *cis-*9 18:1 a different elongase [39] is required. Therefore, the negative correlation may indicate competition between enzymes for the same substrate.

**Figure 2:**
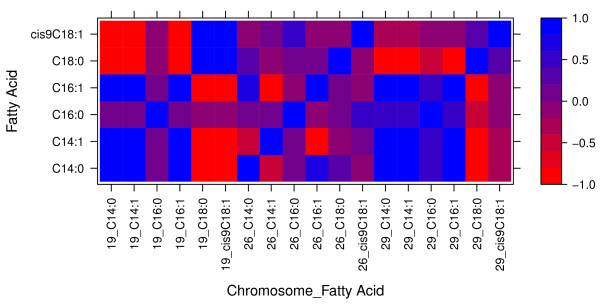
**Within 1-Mb region correlations of direct genomic breeding values for C14:0, C14:1, C16:0, C16:1, C18:0, and cisC918:1 fatty acids on a beef meat basis.** Regions are identified by chromosome number on the X-axis. The Y-axis represents the fatty acids for the same 1-Mb region on the X-axis.

The second correlation pattern involves the region on chromosome 26 (26_21), which harbors SCD. Correlations were in general lower than the ones obtained in the previous two regions. The within region correlation between the 14:0, 16:1 and 18:0 were all strong and positive. Weaker positive correlations were also observed with 16:0. However, the correlations of DGV for those fatty acids with 14:1 and *cis*9 18:1 were negative. Figure 2 summarize the within regions correlations among the same fatty acids on the beef meat basis. The same patterns of correlations were obtained on the beef basis as those obtained for fat percentage basis except for 16:0 (at all three windows) and *cis*9 18:1 (at 26_21 and 29_18 windows) where no QTL was detected on these regions for these fatty acids on the beef basis analysis (Table 6).

Patterns of correlations illustrate how the selection to change fatty acid composition of fat could have a differential effect depending upon the region that is affected by selection. Thus, the use of genomic information creates an opportunity for a more precise selection by using specific regions information rather than pedigree based selection. On the other hand, we have been assuming that the observed correlations are due to pleiotropic effects, which might not be the case. To what extent the correlations are due to selection for increased marbling in the Angus population is unknown.

## Conclusion

This study is the first genome selection and genome wide association analyses for fatty acid composition in American Angus sired cattle. Fatty acid composition is of paramount importance due to their role in cardiovascular health. The genetic dissection of fatty acid composition could lead to a better understanding of the molecular mechanisms that control fatty acid content in meat. We utilized a large Angus-sired population to calculate genomic breeding values of individual animals and to identify genomic regions harboring genetic variation associated with fatty acid composition. Molecular markers were able to account for between 6 and 57% of the observed variance in an individual fatty acid. In addition, the accuracy of the DGV (measured as simple correlations between DGV and phenotype) ranged from -0.06 to 0.57. Furthermore, we identified 57 1-Mb windows with a posterior probability of inclusion (> 0.90) that harbor genetic variation associated with individual fatty acid content. This large number of genomic regions might indicate the presence of an elaborate molecular mechanism that control fatty acid content in skeletal muscle. In addition, the correlation of DGV among the different fatty acids within specific genomic regions might help to articulate the genetic correlations between any two traits. Taken together these results provide the most comprehensive evaluation of the genetic mechanisms that control fatty acid composition in skeletal muscle.

## Methods

All animal work was approved by the Iowa State University Animal Care and Use committee before the conduction of this study.

### Genotype and Phenotype data

A total of 2,177 Angus-sired calves sired by 134 Angus sires were genotyped with the BovineSNP50 BeadChip (Illumina, San Diego, CA). Sixty-seven animals that had incomplete phenotype or fixed effect information were removed, leaving 2,110 animals represented by bulls (n = 500), steers (n = 1,210), and heifers (n = 400), born between 2002 and 2008.

Production characteristics and additional detail of the sample collection and preparation of these cattle were reported previously [40]. After external fat and connective tissue were removed, the 1.27-cm steaks were freeze ground in liquid nitrogen to produce a powder that was analyzed for fatty acid composition. Total lipid was extracted with a chloroform and methanol (2:1, vol:vol) mixture and then quantified [41]. The individual lipid spots were derivatized to methyl esters with acetyl chloride in methanol prior to gas chromatography for determination of fatty acid composition. Fatty acid methyl esters (FAME) were analyzed by gas chromatography (model 3400, Varian, Palo Alto, CA) using a Supelco SP-2380 column (30 m × 0.25 mm i.d. × 0.20 μm film thickness) and a flame ionization detector. The column started at a temperature of 100°C and was ramped up to 170°C at a rate of 2°C per minute, followed by an increase to 180°C at 0.5°C per minute and to 250°C at 10°C per minute. The total running time was 62 min. The temperature of the injector was programmed to increase from 68°C to 250°C at a rate of 250°C per minute. The detector was maintained at 220°C.

The phenotypic observations on fatty acid composition were used as response variables to estimate marker effects for each fatty acid separately. In total, 49 fatty acid traits were analyzed in this study. Each trait was measured in two different ways: 1) beef basis = weight of a given fatty acid, g×10^-5^, in 1 gram meat, 2) fat percent = weight of a given fatty acid in relation to total extracted fatty acid times 100. The individual fatty acids analyzed were: 10:0 (number of carbon atoms : number of unsaturated bonds), 12:0, 13:0, 14:0, 14:1, 15:0, 16:0, 16:1, 17:0, 17:1, 18:0, 18:1c9, 18:1c11, 18:1c12, 18:1c13, 18:1t6pt9, 18:1t10pt11, 18:1t12, 18:1t15, 18:2, 18:3n3, 18:3n6, 20:0, 20:1, 20:2, 20:3n3, 20:3n6, 20:4, 20:5, 22:0, 22:1, 22:4, 22:5, 22:6, 23:0, 24:0, CLAc9t11, and CLAt10c12. Medium chain fatty acids (MCFA) were the sum of 12:0 and 13:0. Long chain fatty acids (LCFA) were the sum of all fatty acids with 14 or more carbons. MUFA, PUFA and SFA were the sum of all monounsaturated, polyunsaturated and saturated fatty acids, respectively. A polyunsaturated to saturated fat index was calculated (PUFA/SFA). A saturation index was calculated as the sum of (14:0 + 16:0) divided by all fatty acid, (14:0+16:0)/All. In addition to fatty acid composition data, atherogenic index (AI) was calculated as proposed by Ulbricht and Southgate [42] as shown below:

$$\mathrm{AI}=\frac{4\cdot C14:0+C16:0}{\sum \mathrm{MUFA}+\sum \mathrm{PUFA}4}$$

The omega-3 (n3) and omega-6 (n6) fatty acids were the sum of 18:3n3 and 20:3n3, or 18:3n6 and 20:3n6, respectively. Also, an omega-3 to omega-6 ratio (n3/n6) was calculated.

### Statistical model

In this study, all 53,367 SNP markers were used as predictors with fatty acid phenotypes as response variables to estimate SNP effects. The “BayesB” method [43] that fits a mixture model where non-zero SNP effects are drawn from distributions with marker specific variance and some known fraction of markers (π) have zero effect was used to estimate marker effects for genomic predictions. For each trait the following model was fit to the estimate marker effects:

y=Xb+Zu+e,

where **y** is the vector of observations for a particular fatty acid trait; **b** is the vector of fixed effects including population mean, contemporary group (defined as feed location-harvest date-sex), and covariates including subcutaneous fat thickness at 12^th^ rib, longissimus muscle area at 12^th^ rib, hot carcass weight, and the amount of chemically extracted fat; **u** is a vector of random marker effects, where element j of **u** has $\sigma_{u_{j}}^{2}>0$ (with probability 1 - π) or $\sigma_{u_{j}}^{2}=0$ (with probability π) as described by [44]; **X** and **Z** are design matrices which relate phenotypic observations to fixed and marker effects, respectively, with each element of **Z** representing allelic state (i.e., number of B alleles from the Illumina A/B calling system); and **e** is the vector of random residuals ~N(0, $\sigma_{e}^{2}$). In this study, parameter π was set to 0.999 for all analyses as high π values were estimated for fatty acid traits in preliminary analyses using BayesCπ method [44]. MCMC methods with 41,040 iterations were used to obtain estimates of marker effects and variances as the posterior means of the corresponding sampled values after discarding the first 1,000 samples to allow for burn-in. In preliminary analyses, the BayesC method [45], which has been shown to be less sensitive to prior assumptions than BayesB [44], was first fitted using prior genetic and residual variances equal to half of total phenotypic variance of each trait and π=0.95 to obtain posterior estimates of genetic and residual variances for constructing priors of genetic and residual scale parameters for BayesB, and to estimate the heritabilities (as the ratios of posterior means of genetic variances over the posterior phenotypic variances) of fatty acid traits.

The DGV for individual i was derived by multiplying the number of copies of B alleles by their corresponding posterior mean SNP effect, and summing these values over all k marker loci:

$$\mathrm{DG}V_{i}=\sum_{j=1}^{k}z_{\mathrm{ij}}{\hat{u}}_{j}$$

where DGV_i_ is the DGV for individual i, z_ij_ is the marker genotype of individual i for marker j, and ${\hat{u}}_{j}$ is the posterior mean effect of marker j obtained from the 40,000 post burn-in samples. Estimated effects of markers within each 1-Mb window (defined by the UMD3.1 assembly) were used every 40^th^ iteration to compute genomic breeding values of all animals for every window. The variance of DGV for any particular window (across all animals) were used to compute the genetic variance of that window. Unmapped markers were considered as an extra window. Posterior probability of inclusion (PPI) for a given window, which is the proportion of samples in which at least one SNP from a given window was included in the model with a non-zero effect, was used for significance testing [46]. A window with PPI > 90% (across 1,000 samples obtained from 40,000 post burn-in samples) was selected as a window containing (or being) a QTL. The PPI has close connections with frequentist approaches that control the false discovery rate [47]. All analyses were performed using GenSel software [48].

Estimates of the proportion of genetic variation explained by each 1-Mb window obtained from the genome-wide association study was plotted against genomic location using SNPLOTz v.1.52 [49]. Individual 1-Mb that explained the largest proportion of genetic variation were then visualized in GBrowse [50] for detailed inspection of the chromosomal region containing the 1-Mb window. Gene searches were performed for these genomic regions with the highest genetic variances.

### Accuracies of DGV

A cross-validation strategy was applied to estimate the accuracies of DGV for traits that may be of interest for breeding. First, the genotyped animals were divided into 6 unequally sized mutually exclusive groups using K-means clustering whereby genomic relatedness was increased within each group and decreased between each of the groups. In this way the detection of true linkage disequilibrium is favored versus just family linkage. Two resultant small groups were combined together to make a single, fifth group. The method of VanRaden et al. [51] was used to construct a genomic relationship matrix between genotyped animals. The Hartigan and Wong [52] algorithm, implemented using R [53] was used for K-means clustering based on a difference matrix obtained from the genomic relationships among the genotyped animals. Details concerning K-means clustering for assigning animals to groups are in Saatchi et al. [54].

Second, a training analysis was undertaken whereby the data excluded one group to train on the remaining groups to estimate marker effects, which then were used to predict DGV of individuals from the omitted group (validation set). This analysis resulted in every animal having its predicted DGV obtained without using its own phenotype nor those of close relatives in training. For each trait, the realized accuracy of DGV was calculated as the pooled correlations between DGV and phenotypes in validation groups divided by the square root of trait heritability.

### Correlation of within 1-Mb region DGV

The DGV for each of three important 1-Mb windows (19_51, 26_21 and 29_18), which harbor the candidate genes FASN, SCD and THRSP, respectively, were calculated for C14:0, C14:1, C16:0, C16:1, C18:0 and *cis-*9 C18:1 fatty acids (those involved in *de novo* synthesis and other abundant fatty acids that are generated by further elongation and desaturation) on both fat percent and beef meet bases. The correlations between DGV for a given 1-Mb window were estimated for each pair of fatty acids using posterior mean of covariances and relevant variances to gain an insight into possible pleiotropic effects of QTL regions associated with these fatty acids.

### Availability of supporting data

All association results have been deposited in the AnimalQTLdb (http://www.animalgenome.org/cgi-bin/QTLdb/BT/qabstract?PUBMED_ID=ISU0064).

### Endnote

^a^This research was supported by Zoetis Animal Genetics.

## Abbreviations

AI: Atherogenic index; CLA: Conjugated linoleic Acid; DGV: Direct genetic value; LCFA: Long chain fatty acid; MCFA: Medium chain fatty acid; MCMC: Markov chain monte carlo; MUFA: Mono-unsaturated fatty acid; PPI: Posterior probability of inclusion; PUFA: Polyunsaturated fatty acids; QTL: Quantitative trait loci; SFA: Saturated fatty acid.

## Competing interests

The authors declare they have no competing interests.

## Authors’ contributions

JMR, DCB, RGT and DG conceived of the experiment and wrote the paper. MSM, MD, JS, and MS collected samples, measured fatty acids, analyzed the data and contributed to the writing of the paper. CD has contributed to the analysis and writing of the paper. All authors read and approved the final manuscript.
